# Dr. Ash

**Published:** 1829-05

**Authors:** 


### OBITUARY.

### DR. ASH.

We have to record the death of Dr. Ash, at his house, in Foley place, on Sunday last, the result of a debilitating state of health, which had long incapacitated him for the active duties of his profession. He was a Fellow of the Royal College of Physicians, and of the Royal Society, and was educated in medicine under the auspices of the celebrated Dr. Ash, his uncle, the founder of the General Hospital, at Birmingham, and whose full-length portrait, by Sir Joshua Reynolds, adorns the board-room of that charity. After spending the portion of time prescribed by his election to a travelling fellowship of Oxford abroad, and principally in Germany, he settled in London as a physician in private practice, which he continued to within a few months of his decease, at the age of 65, greatly weakened and depressed in his physical, but not the least in his *mental* powers.

As a public character, Dr. Ash was little known beyond a select circle of friends, chiefly of the literary and scientific class, by whom he was highly and universally esteemed, as well on account of his strict moral qualities, as his extensive intellectual attainments.

Endowed largely with various stores of knowledge, beyond what are usually acquired in his profession, his habits were yet inobtrusive and unassuming, and his disposition and manners were not well calculated for contention with his brethren in the pursuit of a large and first-rate metropolitan practice, although none probably excelled him in sound professional knowledge, skill, and judgment. In early life he was the intimate friend of Humboldt, and formed an extensive acquaintance with the German schools and professors; in the literature, philosophy, and medical sciences of which he was deeply learned. We have heard it *confidently* asserted, that the burlesque German tragedy, and some other witty effusions, ridiculing German sentiment and manners, usually ascribed to the late Mr. Canning, were written by the subject of this memoir.

As an author, Dr. Ash is unknown to the public, although in physiology and chemistry, his experimental researches have been very numerous; and his manuscript notes, we have reason to believe, are generally referrible to that extraordinary variety and accuracy of information which he was known to possess in universal literature and science, and which characterized him as a gentleman and a scholar of no common stamp. United to a strong memory, he possessed peculiar talents for the acquirement and communication of knowledge, and which were *most effectually and successfully* applied to the domestic education of a large family of sons, without at all interfering with his medical duties, and general pursuits. But this habit was apparently the principal cause of his indulging very little in *social* intercourse with the world, which is generally considered so essentially requisite to ensure fame and eminence in his profession in the present age. Dr. Ash's merits are to be measured by a different scale; and among those who knew him well, we venture to say, his memory will long be cherished with a degree of estimation undiminished by his rare appearance in the *luxurious* circles of London society.—*Med. Gazette.*

